# New chemistry for enhanced carbon capture: beyond ammonium carbamates

**DOI:** 10.1039/d0sc06059c

**Published:** 2020-12-07

**Authors:** Alexander C. Forse, Phillip J. Milner

**Affiliations:** Department of Chemistry, University of Cambridge Cambridge CB2 1EW UK acf50@cam.ac.uk; Department of Chemistry and Chemical Biology, Cornell University Ithaca New York 14853 USA pjm347@cornell.edu

## Abstract

Carbon capture and sequestration is necessary to tackle one of the biggest problems facing society: global climate change resulting from anthropogenic carbon dioxide (CO_2_) emissions. Despite this pressing need, we still rely on century-old technology—aqueous amine scrubbers—to selectively remove CO_2_ from emission streams. Amine scrubbers are effective due to their exquisite chemoselectivity towards CO_2_ to form ammonium carbamates and (bi)carbonates, but suffer from several unavoidable limitations. In this perspective, we highlight the need for CO_2_ capture *via* new chemistry that goes beyond the traditional formation of ammonium carbamates. In particular, we demonstrate how ionic liquid and metal–organic framework sorbents can give rise to capture products that are not favourable for aqueous amines, including carbamic acids, carbamate–carbamic acid adducts, metal bicarbonates, alkyl carbonates, and carbonic acids. These new CO_2_ binding modes may offer advantages including higher sorption capacities and lower regeneration energies, though additional research is needed to fully explore their utility for practical applications. Overall, we outline the unique challenges and opportunities involved in engineering new CO_2_ capture chemistry into next-generation technologies.

## Abstract 2

New pathways for carbon capture and sequestration are needed to tackle the challenge of rising anthropogenic carbon dioxide emissions.

## Graphical abstract

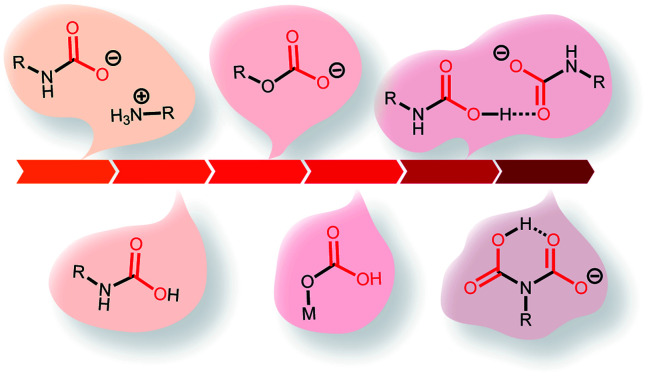

## Introduction

Rising atmospheric levels of carbon dioxide (CO_2_) are the major contributor to global climate change, with annual emissions approaching 40 billion tonnes.^1^ Nearly two-thirds of anthropogenic CO_2_ emissions result from the combustion of fossil fuels, including coal and natural gas, for the global production of electricity.^1^ In addition, CO_2_ emissions are an inevitable by-product of other industrial processes, including the production of cement, steel, and natural gas.^1^ As a result, new technologies are needed to mitigate emissions from these industrial point sources during the gradual transition to cleaner fuels and building materials. One such proposed technology is carbon capture and sequestration or utilization, in which CO_2_ is selectively removed from low-concentration emission streams (4–15% CO_2_) prior to its permanent storage underground or conversion into more valuable products.^2^

**Fig. 1:**
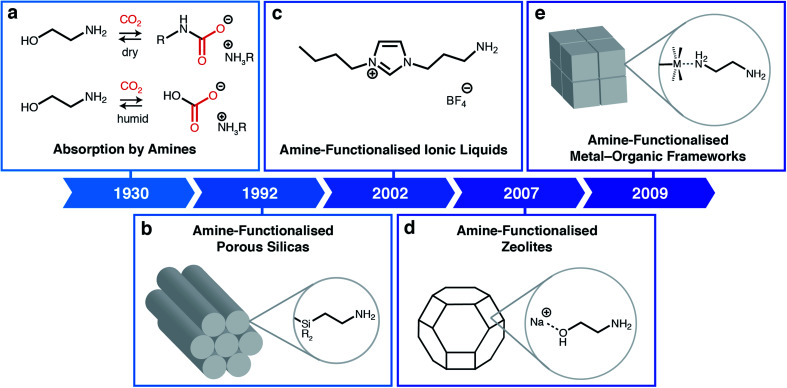
Classes of amine-based materials for CO_2_ capture. (a) Traditional amine chemistry for CO_2_ absorption *via* the formation of ammonium carbamates under dry conditions and ammonium (bi)carbonates under humid conditions.^3^ (b) Amine-functionalised porous silicas, where amines are either impregnated into or covalently attached to silica supports.^12^ (c) Amine-functionalised ionic liquids.^13^ (d) Post-synthetically amine-functionalised zeolites.^14^ (e) Post-synthetically amine-functionalised metal–organic frameworks.^15^

Building upon technology developed in the 1930s to purify crude natural gas, many have shown that aqueous amine scrubbers are currently the most technology-ready sorbents for CO_2_ capture from flue emissions on large scale (Fig. 1a).^3^ Aqueous amine scrubbers are effective because amines react selectively with CO_2_ to produce carbamic acid intermediates, which rapidly react with a second equivalent of amine to produce ammonium carbamates; under aqueous conditions, ammonium carbamates and carbamic acids can further react with water to produce ammonium (bi)carbonates.^4^ The captured CO_2_ is then desorbed using heat and/or vacuum (temperature and/or vacuum swing), thereby regenerating free amines. Over the last ninety years, there has been significant optimisation of the amine structure to maximize working capacities (*i.e.* the usable amount of CO_2_ captured in an actual process) while minimising regeneration energies (*i.e.* the total energy input needed to heat the material and desorb CO_2_).^5^ However, aqueous amine scrubbers are still faced with several challenges, including: (1) low capacities (<3 mol CO_2_ per kg solution or <15 wt%) due to dilution of the corrosive amines with water;^6^ (2) poor oxidative stability of amines towards O_2_; and (3) degradation in the presence of contaminants such as SO_2_, which reacts with amines similarly to CO_2_.^7^ In addition, one aspect of aqueous amine scrubbers has remained largely constant: the products of their reaction with CO_2_.^8^ This restriction generally leads to high regeneration energies (≥2.4 MJ kg^−1^ CO_2_) and CO_2_ desorption temperatures (>100 °C), greatly increasing the cost of carbon capture from flue emissions.^9–11^

## Amine-based materials for CO_2_ capture

One promising avenue to overcome the challenges associated with CO_2_ capture by aqueous amine scrubbers is to employ other types of sorbents, such as porous solids or ionic liquids (ILs). Porous materials such as silicas, carbons, zeolites, metal–organic frameworks (MOFs), porous organic polymers (POPs), and covalent-organic frameworks (COFs), have the potential advantages of higher thermal stabilities and lower heat capacities compared to aqueous amine scrubbers.^16–21^ Likewise, ILs are low-melting ionic salts that offer advantages over aqueous amines including non-volatility (preventing release into the atmosphere) and structural tunability. Although hydrophobic porous solids such as silicon-rich zeolites and carbons are capable of scrubbing CO_2_ from high-concentration streams (*e.g.* crude biogas),^22^ many of these materials cannot remove CO_2_ from humid low-concentration streams such as flue gas emissions.^23^ This limitation arises because CO_2_ and water directly compete for the same physisorption sites in these sorbents. An additional general challenge for porous solid adsorbents that remains to be addressed is their poor thermal conductivity, which complicates adsorbent heating and cooling during adsorption/desorption cycling.

A powerful approach to overcome the poor selectivities of typical sorbents towards CO_2_ under humid conditions is to leverage the favourable reactivity of aqueous amine scrubbers in the form of amine-functionalised sorbents (Fig. 1).^24,25^ Beginning with the first report of amine-functionalised silicas in 1992 (Fig. 1b),^12,24^ a range of amine-functionalized solid adsorbents, including zeolites (Fig. 1d),^14^ MOFs (Fig. 1e),^15,19^ and carbons^26^ have been prepared. Researchers have demonstrated that amine-functionalised porous solids possess the high CO_2_ selectivities native to aqueous amines while generally evidencing improved thermal and chemical stabilities. For example, confining amines within a porous support largely eliminates oxidation pathways that are catalyzed by leached metal ions from the absorption columns.^7,27^ Similarly, ionic liquids (ILs) can also be functionalised with amine groups to achieve high CO_2_ capacities and selectivities without the need for dilution with water (Fig. 1c).^28,29^ Numerous *in situ* spectroscopic studies using solution- and solid-state nuclear magnetic resonance (SSNMR) and infrared (IR) spectroscopy combined with theoretical calculations suggest that in most cases amine-functionalised materials produce similar sorption products as aqueous amine scrubbers, namely, ammonium carbamates under dry conditions^13,30,31^ and, as confirmed recently, ammonium bicarbonates under humid conditions.^32^ As such, the majority of these materials still require high temperatures (>120 °C) to fully desorb CO_2_, resulting in high regeneration penalties.^33^ In addition, amine-functionalised silicas suffer from oxidative degradation by distinct bimolecular pathways,^34^ as well as the irreversible formation of ureas under dry conditions.^35^ Overcoming these fundamental limitations is critical to enabling the widespread adoption of carbon capture technologies.

## New CO_2_ chemisorption pathways in solution and the solid state

An underexplored approach to overcome the fundamental limitations of amine-based materials is not to focus on the development of new materials, but on new chemisorptive pathways for selective carbon dioxide capture. For example, the formation of carbamic acids by CO_2_ capture at amine sites is potentially desirable because it involves reaction with CO_2_ at only a single amine site, increasing the CO_2_ : amine sorption ratio to 1 : 1.^36^ Indeed, unlocking 1 : 1 reaction stoichiometries in general should produce higher gravimetric and volumetric sorption capacities by enabling a higher density of reactive sites within a given volume. Additionally, mechanisms beyond ammonium carbamate formation have been shown to lead to lower CO_2_ desorption temperatures in some cases (see below). Importantly, each combination of CO_2_ partial pressure (*P*) and temperature (*T*) for a given separation (*e.g.* 400 ppm, 25 °C for capture directly from the atmosphere) leads to an ideal differential free energy of sorption (−Δ*G*) for that separation (*e.g.* −19 kJ mol^−1^ for direct air capture), which is critical to maximising sorption capacities while minimising regeneration energies.^41^ New chemisorption pathways should enable more dramatic tuning of the differential enthalpies (−Δ*H*) and entropies (−Δ*S*) of sorption to achieve these optimal values. Last, moving away from amines entirely could lead to adsorbents with improved oxidative stabilities, a recurring challenge associated with amine-based materials, although more work is required to characterize the oxidative stability of promising sorbents.^42^ Here, we highlight examples of new CO_2_ adsorption pathways beyond ammonium carbamates that may ultimately lead to enhanced CO_2_ capture.

**Fig. 2:**
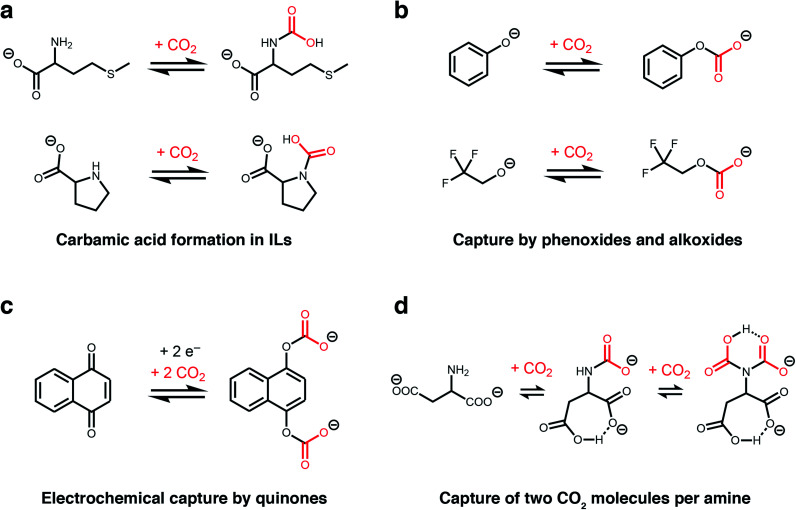
New CO_2_ absorption mechanisms in solution. (a) Proposed formation of carbamic acid in ILs with amine-functionalised anions.^37^ (b) Proposed absorption mechanism by phenoxide and alkoxide ILs.^38^ (c) Proposed mechanism for electrochemical CO_2_ capture by 1,4-naphthoquinone.^39^ (d) Proposed absorption mechanism for an IL with an aspartate dianion.^40^ In all cases, the corresponding cations are omitted for clarity.

The unique, highly-charged environment within ILs makes them an ideal setting to unlock new CO_2_ reactivity. For example, although carbamic acids are normally disfavoured outside of polar aprotic solvents (*e.g.* dimethyl sulphoxide),^36,43^ Schneider, Brennecke, and coworkers found that installing amines onto the anions of amino acid-derived ILs favours CO_2_ capture *via* the formation of carbamic acids stabilized by hydrogen-bonding (Fig. 2a).^37^ This change in mechanism doubled the molar absorption capacity of these ILs compared to those bearing amine-functionalized cations, which operate by the traditional ammonium carbamate mechanism.^13^ In addition, the strong binding of CO_2_ within a proline-derived IL (−Δ*H*_abs_ = 80 kJ mol^−1^) led to nearly complete saturation at low pressures of CO_2_ (<0.1 bar at 25 °C). Therefore, this switch in chemisorption products demonstrates that the local environment of an amine is a crucial design element for controlling its reactivity towards CO_2_.^44^

Following these initial studies, even more unconventional CO_2_ absorption pathways began to emerge in ILs. Building upon previous reports,^45^ Li, Dai, and coworkers demonstrated that amines can be completely bypassed by capturing CO_2_ in ILs bearing alkoxide or phenoxide anions and organic superbase-derived cations, which reversibly capture CO_2_*via* alkylcarbonate formation (Fig. 2b).^38^ Similar to carbamic acids (Fig. 2a), this chemistry gives rise to a 1 : 1 reaction stoichiometry and thus higher gravimetric capacities (up to 20 wt%) compared to traditional IL sorbents (<10 wt%). Importantly, alkoxide-based ILs also possess low viscosities and rapid absorption kinetics (saturation in less than 5 minutes at room temperature), overcoming common challenges that plague traditional amine-functionalized ILs.^38^ Subsequently, Kim and coworkers demonstrated that similar reactivity at oxygen could be achieved in water-lean alcoholamines bearing sterically-hindered amines and that the resulting ammonium alkylcarbonates desorb CO_2_ more readily than ammonium carbamates.^50^

Another route to generate oxyanion nucleophiles for rapid CO_2_ capture *via* carbonate formation is by the electrochemical reduction of quinones, as demonstrated by Hatton and others (Fig. 2c).^39^ Promising results with electrochemically-reduced quinones have been observed in the presence of water and oxygen, although some loss in capacity was observed due to re-oxidation of the nucleophile by oxygen.^51^ This electrochemical approach has subsequently been expanded to other nucleophiles, such as reduced sulphides, suggesting it may be a general strategy to expand the scope of nucleophiles for CO_2_ capture.^52^ An advantage of this approach is that electrochemical regeneration of the quinone (electrochemical swing adsorption) leads to energy savings over traditional temperature or pressure swing processes.

Recent work has revealed that CO_2_ capacities approaching a remarkable 2 : 1 reaction stoichiometry can be accessed in ILs, representing a four-fold increase compared to the traditional ammonium carbamate mechanism (Fig. 2d).^40^ Specifically, Wang and coworkers found that an ionic liquid with an aspartate dianion was able to reversibly bind 1.96 mol CO_2_ per mol IL at 30 °C and 1 atmosphere CO_2_, which was hypothesised to occur *via* two subsequent reactions at a single amine site to form both a carbamate (calculated Δ*E*_abs_ = −69 kJ mol^−1^) and a carbamic acid (calculated Δ*E*_abs_ = −54 kJ mol^−1^).^40^ This proposed absorption pathway was supported by ^13^C NMR measurements as well as density functional theory (DFT) calculations, with the latter ruling out reaction of CO_2_ at the carboxylate groups as proposed for related ILs.^53^ A similar 2 : 1 absorption mode was also evidenced in an earlier organic chemistry study. The observation of a triplet in solution ^15^N NMR studies of selected primary amines in the presence of ^13^CO_2_ and a base confirmed the reaction of 2 CO_2_ molecules with a single amine group at −30 °C.^53^ The high capacity offered by this absorption mode makes it a very attractive target for CO_2_ capture applications.

Although ILs and water-lean solvents represent a unique platform for the discovery of new CO_2_ capture products, they are not without their own challenges. For example, the absorption capacities of most ILs are relatively low (<20 wt%) compared to amine-functionalized solids.^13,28^ In addition, the viscosities of ionic liquids are relatively high and tend to increase upon CO_2_ adsorption (in some cases up to 200-fold), which represents a significant process challenge.^28,54^ While molecular engineering allows access to CO_2_-loaded ILs with viscosities as low as 650 mPa s,^55^ these values are still significantly higher than CO_2_-loaded 30% aqueous monoethanolamine solution (4 mPa s).^56^ Last, the CO_2_/N_2_ absorption selectivities, kinetics, desorption conditions, and long-term cycling stabilities of ILs remain poorly characterized in many cases. Addressing these challenges is critical to advancing the commercial viability of IL-based sorbents.

An emerging alternative approach is to engineer new CO_2_ capture mechanisms within the controlled pore environments of crystalline porous materials, such as MOFs. The arrangement of functional groups in an ordered fashion within the pores of MOFs presents a potential opportunity for unlocking new CO_2_ capture chemistry.

**Fig. 3:**
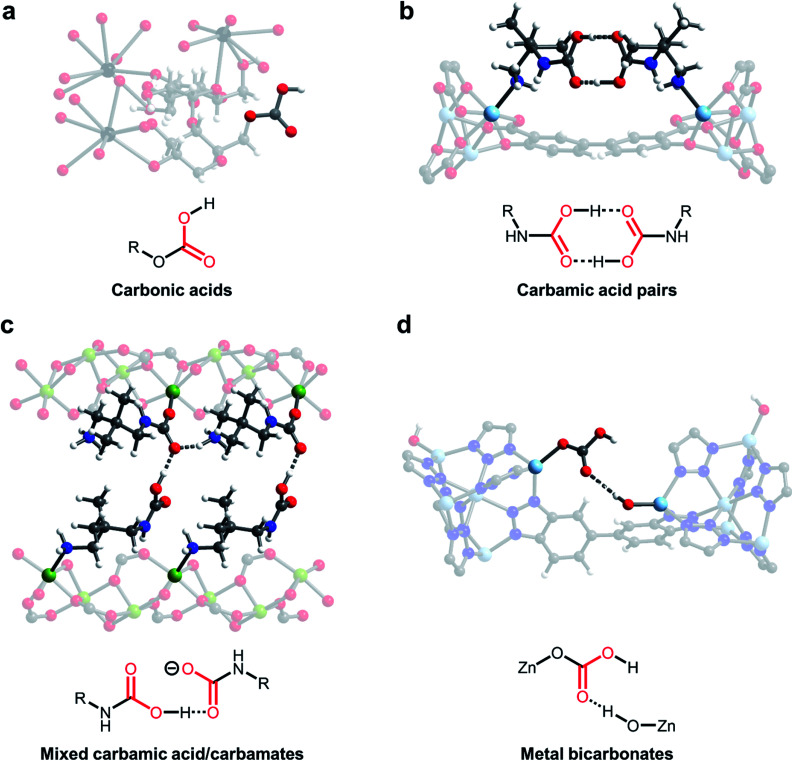
New CO_2_ adsorption mechanisms unlocked in MOFs. (a) Proposed formation of carbonic acids in CD-MOF-2 (CD = cyclodextrin).^46^ (b) Crystallographically confirmed formation of carbamic acid pairs in dmpn–Zn_2_(dobpdc) (dmpn = 2,2-dimethyl-1,3-diaminopropane; dobpdc^4−^ = 4,4′-dioxidobiphenyl-3,3′-dicarboxylate).^47^ (c) Proposed formation of mixed carbamic acids and ammonium carbamates in dmpn–Mg_2_(dobpdc).^48^ (d) Proposed formation of metal bicarbonates in Zn(ZnOH)_4_(bibta)_3_ (bibta^2−^ = 5,5′-bibenzotriazolate).^49^ Gray, white, red, black, dark blue, sky blue, and green spheres correspond to carbon, hydrogen, oxygen, rubidium, nitrogen, zinc, and magnesium, respectively.

One of the earliest demonstrations of CO_2_ chemisorption in MOF adsorbents involved CD-MOFs (Fig. 3a; CD = γ-cyclodextrin).^46,57^ These MOFs demonstrate strong adsorption of CO_2_ at low partial pressures (<2 mbar), leading to excellent CO_2_/CH_4_ selectivity (estimated to be >3000) in this regime.^46^ Using SSNMR measurements, the authors proposed the formation of carbonic acids or alkylcarbonates; however, the exact chemisorption pathway in this material remains unclear. Nonetheless, the strong bonding of CO_2_ in CD-MOF-2 (>1 mmol CO_2_ per g MOF adsorbed at 10 mbar and 30 °C) makes this a promising potential material for flue gas capture applications. Analysis of the thermodynamics of CO_2_ chemisorption in this material by calorimetry revealed a moderate enthalpy of adsorption at intermediate loadings (−Δ*H*_ads_ = 65 kJ mol^−1^), enabling easier desorption of CO_2_ from the strong-binding sites compared to amines.^58,59^ However, the poor water stability of these MOFs necessitates the translation of this chemisorption mechanism to more stable materials for practical applications.^46^

Carbamic acids have long been invoked as intermediates and products upon CO_2_ capture in amine scrubbers,^4^ amine-functionalized silicas^30,60^ and amine-functionalized MOFs,^61,62^ as suggested by NMR and IR spectroscopies. For example, Ho and coworkers found that hydrazine-functionalized variants of the MOF Mg_2_(dobdc) (dobdc^4−^ = 2,5-dioxido-1,4-benzenedicarboxylate) exhibit incredibly strong and selective binding of CO_2_ (3.89 mmol g^−1^ at 25 °C and 0.4 mbar of CO_2_), which they ascribe to highly favourable carbamic acid formation (−Δ*H*_ads_ = 90 kJ mol^−1^) within the framework pores.^62^ However, until recently there remained little crystallographic evidence for this elusive adsorption product in the solid state. Long and coworkers identified variants of the MOF M_2_(dobpdc) (dobpdc^4−^ = 4,4′-dioxidobiphenyl-3,3′-dicarboxylate) functionalised with the diamine 2,2-dimethyl-1,3-diaminopropane (dmpn) as promising adsorbents for post-combustion CO_2_ capture owing to their exceptional hydrothermal and oxidative stability (Fig. 3b and c).^47,48^ Exposure of single crystals of dmpn–Zn_2_(dobpdc) to 1 bar of CO_2_ induced the formation of carbamic acid pairs bridging two adjacent amine sites in the framework, as confirmed by SCXRD and SSNMR (Fig. 3b).^47,48^ In this structure, the normally disfavoured formation of carbamic acids is facilitated by well-defined hydrogen-bonding interactions, corroborated by the presence of strong ^1^H_(COOH)_⋯^13^C correlations in 2-dimensional SSNMR experiments. Notably, carbamic acid pairs were actually predicted computationally in related frameworks before they were observed experimentally.^63^

Building upon this work, the same group demonstrated that dmpn–Mg_2_(dobpdc) chemisorbs CO_2_ by another distinct pathway: the formation of both ammonium carbamates and carbamic acids (Fig. 3c).^48^ In-depth DFT calculations and 2-dimensional SSNMR experiments support the formation of ammonium carbamate chains that interact with carbamic acids *via* hydrogen-bonding in this material. The advantage of this mechanism lies in its high enthalpy of adsorption (Δ*H*_ads_ = −74 kJ mol^−1^) coupled with a large entropic penalty (−Δ*S*_ads_ = 204 J mol^−1^ K^−1^), which reduces the temperature required to desorb CO_2_ in a temperature-swing adsorption process to <100 °C, potentially enabling adsorbent regeneration with low-grade steam.^47^ These thermodynamic parameters enable adsorbent regeneration with an estimated energy of 2.5 MJ kg^−1^ CO_2_, comparable to the best-in-class aqueous amine scrubbers such as Mitsubishi KS-1 (2.4 MJ kg^−1^ CO_2_).^10,64^ Therefore, this finding highlights the potential to overcome thermodynamic trade-offs of carbon capture processes by tuning the adsorption pathway. In addition, this adsorption mode leads to faster adsorption kinetics than ammonium carbamate formation in related materials and a high non-competitive CO_2_/N_2_ selectivity (880) under the conditions relevant for CO_2_ capture from coal flue emissions (150 mbar CO_2_, 750 mbar N_2_, 40 °C).^65^

A further promising avenue to unlock new selective CO_2_ capture reactivities in porous materials is to look to nature for inspiration. For example, carbonic anhydrase enzymes are responsible for the transport of CO_2_ in the human body. Many members of this family operate by the reversible reaction of a zinc-hydroxide species (Zn–OH) with CO_2_ to form a zinc-bound bicarbonate species (Zn–OCO_2_H).^66^ In an early study, Zhang and coworkers demonstrated that high-valent monodentate metal hydroxides in the water-stable MOFs Mn^II^Mn^III^(OH)Cl_2_(bbta) and [Co^II^Co^III^(OH)Cl_2_(bbta)] (bbta^2−^ = dihydrobenzo[1,2-*d*:4,5-*d*′]bis([1,2,3]triazolate)) strongly bind CO_2_ with high CO_2_/N_2_ selectivity (>250), even under humid conditions.^67^ These materials exhibit highly exothermic capture of CO_2_ at low loadings (−Δ*H*_ads_ > 100 kJ mol^−1^), necessitating regeneration using flowing N_2_ at 85 °C (simulating a temperature-vacuum swing process). A regeneration energy of 2.7 MJ kg^−1^ CO_2_ was calculated for [Co^II^Co^III^(OH)Cl_2_(bbta)], which is comparable with best-in-class aqueous amines. Closely mimicking the mechanism of carbonic anhydrase enzymes, Wade and coworkers subsequently found that Zn–OH centers in the air-stable MOF Zn(ZnOH)_4_(bibta)_3_ (bibta^2−^ = 5,5′-bibenzotriazolate) strongly bind CO_2_ to form metal-bound bicarbonates with adsorption capacities of 2.2 mmol g^−1^ at 27 °C and 0.4 mbar of CO_2_, suitable for direct air capture (Fig. 3d).^49^ Interestingly, DFT calculations suggest that these metal-bound bicarbonates hydrogen-bond with adjacent Zn–OH centers, stabilizing the adsorption product (−Δ*H*_ads_ = 71 kJ mol^−1^) and leading to steep uptake of CO_2_ at low pressures. Dincă and coworkers demonstrated a similar bioinspired approach to CO_2_ capture in (Zn_5_(OH)_4_(btdd)_3_) (btdd = bis(1,2,3-triazolo[4,5-*b*],[4′,5′-*i*])dibenzo[1,4]dioxin), a hydroxide-substituted variant of the MOF MFU-4*l*.^68^ This framework was found to exhibit stronger CO_2_ binding (−Δ*H*_ads_ = 81 kJ mol^−1^) compared to Zn(ZnOH)_4_(bibta)_3_, albeit with a lower adsorption capacity at low pressures (0.9 mmol g^−1^ at 25 °C and 23 mbar of CO_2_). In all of these studies, the formation of metal-bound bicarbonates was validated primarily by *in situ* IR spectroscopy and DFT calculations. Subsequent work by Wade and coworkers has highlighted the importance of metal identity on CO_2_ adsorption in M–OH MOFs, unveiling a potential handle for tuning the thermodynamics of chemisorption.^69,70^ In a similar vein, Wang and Lackner have found that hydroxide-functionalised ion-exchange membranes are promising for energy-efficient moisture swing sorption processes, demonstrating that ammonium cations can be used as an alternative to metal ions to prepare hydroxide-rich materials.^71^ The capture of CO_2_ with oxygen-based nucleophiles in both adsorbents (Fig. 3a and d) and solution (Fig. 2b and c) represents a promising solution to overcome the inherent limitations of amine-functionalised materials; however, more work is required to map out the stability of these materials and their performance under realistic conditions.

The vast majority of CO_2_ capture processes discussed above operate *via* the addition of nitrogen- or oxygen-based nucleophiles to CO_2_. Recently, the range of nucleophiles that can reversibly react with CO_2_ has been expanded to include electrochemically-generated sulphides,^52^ frustrated Lewis pairs,^72^ and *N*-heterocyclic carbenes,^73^ among others.^74^ In addition, electrochemistry has emerged as a powerful tool to expand the scope of CO_2_ capture processes. For example, Hamelers and coworkers have shown that capacitive charging and migration of bicarbonate/carbonate ions through ion exchange membranes can drive a CO_2_ capture process with a low energy requirement of 40 kJ mol^−1^ CO_2_ captured.^75^ Similarly, Landskron and coworkers have developed a related process in which a supercapacitor device reversibly adsorbs CO_2_ (<0.1 mmol g^−1^), although the exact adsorption mechanism remains unclear.^76^ Finally, electrochemically driven pH swings are also being investigated as a new energy-efficient CO_2_ capture strategy.^77^ These recent directions represent an exciting opportunity to unlock new carbon capture chemistry.

## Opportunities and challenges for next-generation CO_2_ capture

**Fig. 4:**
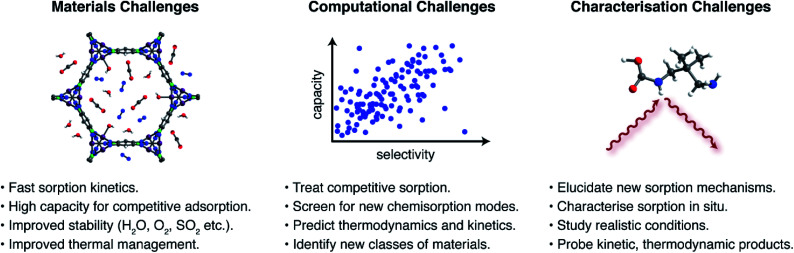
Grand challenges for next-generation sorbents for CO_2_ capture.

The foregoing examples highlight the unique opportunities offered by new CO_2_ capture pathways. Potential advantages of new sorption modes include lower regeneration energies, higher working capacities, and access to a wider range of sorption enthalpies compared to traditional ammonium carbamate formation. Despite this promise, there remains a great need for additional research to assess the application of novel sorption pathways in industrial processes. Key materials challenges (Fig. 4) that remain critically underappreciated include: (i) rapid sorption kinetics, (ii) large working capacities under realistic mixed-gas conditions, (iii) sufficient material stability to survive long-term exposure to reactive contaminants in target gas streams, such as water, oxygen, sulphur dioxide, and hydrogen sulphide, among others depending on the process,^23,24^ (iv) adsorbent development and structuring to overcome issues with low thermal conductivities and heat management during highly exothermic sorption processes, and (v) sustainable and scalable materials synthesis. As an example, the hydroxide-based MOF [Co^II^Co^III^(OH)Cl_2_(bbta)] has a promising working capacity and regeneration energy for a flue gas capture process, but its stability in the presence of oxygen and other contaminants remains unknown.^67^ Moreover the very large heat of CO_2_ adsorption at low loadings (−Δ*H*_ads_ > 100 kJ mol^−1^) suggests that heat management will be an important challenge for this material. Promising strategies to aid heat management include the development of structured hollow fibre adsorbents and the design of carbon-based materials that have inherently larger thermal conductivities.^78^

Exploration of entirely new CO_2_ capture pathways beyond those described in this perspective should also lead to further advances. Recent studies have highlighted the promise of large-scale computational screenings in the search for new CO_2_ capture materials. For example, Smit and coworkers recently reported the screening of over 300 000 theoretical MOFs and identified classes of physisorption sites, termed “adsorbophores”, that endow high CO_2_ selectivities to frameworks.^79^ The guided synthesis of optimised materials for operation under humid conditions was achieved by selecting candidates with hydrophobic adsorbophores to maximize the adsorption of CO_2_ under humid conditions. However, the capacities and CO_2_/N_2_ selectivities reported for best-in-class physisorbents are typically lower than those reported for chemisorptive materials. Similar computational screens to predict chemisorption—for example, using a higher level of theory to account for bond-breaking and – forming processes—remain rare but have the potential to be transformative.^63,80^ Similarly, calculations that can predict chemisorption thermodynamics under realistic mixed gas conditions should lead to promising materials for real-world applications.^79^ Due to the complex processes inherent to chemisorption, an additional challenge for computational analyses is to predict transition states relevant to sorption kinetics. A promising strategy to address these computational challenges may be to use machine learning to guide the search for new chemisorbent materials.^81^

In order to elucidate and ultimately build upon new CO_2_ capture chemistry, advanced characterisation methods are also needed. These methods serve to both validate and discover new chemisorption products when unexpected sorption properties arise. Recent years have seen significant advances in the characterisation of CO_2_ capture pathways through *in situ* spectroscopic and X-ray diffraction experiments.^30,32,47,48^ These experiments must now be adapted to study conditions that more closely mimic envisaged industrial applications and, in particular, must address mixed gas conditions rather than pure CO_2_.^32,48^ Furthermore, experiments must not be restricted to studying static/equilibrium conditions and should probe the dynamic conditions associated with practical sorption processes.

## Conclusions

New CO_2_ sorption pathways such as those recently uncovered in appropriately-functionalised ILs and MOFs may offer improved performance for CO_2_ capture compared to traditional sorbents, including higher capacities and lower regeneration costs. Many of these binding modes do not readily occur in aqueous solution and instead arise from the unique opportunity to precisely install chemical functional groups with a controlled spatial arrangement and carefully tuned local environment. For many of these prospective sorbents, more work is needed to assess their sorption kinetics, selectivities, stabilities, and thermal conductivities. This mechanistically-focused line of sorbent discovery is still in its infancy, and a new generation of computational, analytical, and synthetic chemistry is needed to design transformative materials – and sorption mechanisms – for reducing anthropogenic CO_2_ emissions.

## Conflicts of interest

The authors declare the following competing interest: P. J. M. is listed as an inventor on several patents related to the preparation of metal–organic frameworks for CO_2_ capture.

## Supplementary Material
